# A novel neural network model with distributed evolutionary approach for big data classification

**DOI:** 10.1038/s41598-023-37540-z

**Published:** 2023-07-08

**Authors:** K. Haritha, S. Shailesh, M. V. Judy, K. S. Ravichandran, Raghunathan Krishankumar, Amir H. Gandomi

**Affiliations:** 1 Department of Computer Applications, Cochin University of Science and Technology, Cochin, Kerala India; 2 Department of Computer Science, Cochin University of Science and Technology, Cochin, Kerala India; 3 Department of Mathematics, Amrita School of Physical Sciences, Amrita Vishwa Vidyapeetham, Coimbatore, India; 4 Information Technology Systems and Analytics Area, Indian Institute of Management Bodh Gaya, Bodh gaya, Bihar 824234 India; 5 Faculty of Engineering and Information Technology, University of Technology Sydney, Sydney, NSW Australia; 6 University Research and Innovation Center (EKIK), Óbuda University, Budapest, 1034 Hungary

**Keywords:** Computational science, Computer science, Information technology

## Abstract

The considerable improvement of technology produced for various applications has resulted in a growth in data sizes, such as healthcare data, which is renowned for having a large number of variables and data samples. Artificial neural networks (ANN) have demonstrated adaptability and effectiveness in classification, regression, and function approximation tasks. ANN is used extensively in function approximation, prediction, and classification. Irrespective of the task, ANN learns from the data by adjusting the edge weights to minimize the error between the actual and predicted values. Back Propagation is the most frequent learning technique that is used to learn the weights of ANN. However, this approach is prone to the problem of sluggish convergence, which is especially problematic in the case of Big Data. In this paper, we propose a Distributed Genetic Algorithm based ANN Learning Algorithm for addressing challenges associated with ANN learning for Big data. Genetic Algorithm is one of the well-utilized bio-inspired combinatorial optimization methods. Also, it is possible to parallelize it at multiple stages, and this may be done in an extremely effective manner for the distributed learning process. The proposed model is tested with various datasets to evaluate its realizability and efficiency. The results obtained from the experiments show that after a specific volume of data, the proposed learning method outperformed the traditional methods in terms of convergence time and accuracy. The proposed model outperformed the traditional model by almost 80% improvement in computational time.

## Introduction

Artificial Neural Networks are built from a collection of connected units or nodes known as artificial neurons, which are roughly modelled after the neurons in the biological brain. Warren McCulloch, Walter Pitts, and Donald Hebb introduced the concept of an artificial neural network (ANN) in the 1940s^1^. In the beginning, it was a means of simulating intelligent behavior by modelling the interconnected circuits of neurons in the brain. Nodes in an ANN include an input layer, hidden layer(s), and an output layer. There are connections between each node, or artificial neuron, and each one has a threshold and weight associated with it. Any node whose output exceeds the predefined threshold value is activated and begins providing data to the subsequent layer of the network. If this condition is not met, no data is sent to the next network layer.

When training a neural network, the standard approach involves randomly selecting a starting point and then following the gradient till reaching the top of the hill. This method is known as gradient decent algorithm. For objective functions with a single peak, such as the cost function in linear regression, this method is highly effective and efficient. However, in most real-world scenarios, we have a tremendously complicated problem described as landscapes made up of many peaks and valleys. Such algorithms fail due to their intrinsic tendency to become stuck at the local optima. Another flaw is the inefficiency of differential operation. Hidden layers in multilayer networks typically employ sigmoid transfer functions. Because they compress an infinite input range into a finite output range, these functions are commonly referred to as “squashing” functions. The defining feature of a sigmoid function is that its slope tends to zero as the input is big. For example, if you are using steepest descent to train a multilayer network with sigmoid functions, you might run into trouble if the magnitude of the gradient changes less and less as the input size grows; this would result in increasingly minor adjustments to the weights and biases, even though they are still well off their optimal values. In contrast, Genetic Algorithms (GAs) are potent optimization techniques that provide a fast search strategy for a large problem space. Replacing the gradient descent with a GA might be useful in avoiding the above-mentioned issues. Also, Genetic Algorithms have many advantages over other global optimization schemes, such as Particle Swarm Optimization (PSO), Ant Colony Optimization (ACO), Simulated Annealing (SA) etc. Genetic algorithms use a population-based approach, allowing for diverse solutions to be explored while converging towards promising regions. This characteristic makes GA well-suited for tackling complex optimization problems, as GA explores the search space extensively, ensuring a diverse set of potential solutions is considered. Also, The parallel nature of GA allows for simultaneous evaluation and evolution of multiple solutions, enabling faster convergence and improved scalability. GA possesses inherent robustness against local optima due to its population-based nature and the mechanisms of selection, crossover, and mutation. This characteristic allows GA to escape from suboptimal solutions and continue searching for better solutions globally. Genetic Algorithms have been successfully applied to a wide range of optimization problems, including feature selection, parameter tuning, and classification tasks in literature.

This study intends to employ GA to train the neural networks. The GA is widely used in many subfields of management science, operational research, and industrial engineering since it is one of the most well-known metaheuristic algorithms. If the iterative processes required by the genetic operators can be implemented in a parallel and distributed computer architecture, GAs would be more effective in solving large-scale optimization issues. In order to harness the power and efficiency of GA evolution when training ANNs, this research suggests incorporating the popular GA into the widely-used Apache Spark distributed computing framework. The major contribution of our work lies in the integration of a neural network model and a distributed evolutionary approach, specifically tailored for big data classification. While both neural networks and evolutionary algorithms have been extensively studied independently, our research presents a novel approach by combining these two domains to enhance the accuracy and scalability of classification tasks involving large datasets using Apache Spark framework.

## Literature review

Neural networks are powerful tools for solving complex systems due to their ability to learn and recognize patterns from data. By training on large datasets, Neural Networks can approximate complex functions, make predictions, optimize processes, and detect anomalies. Their versatility and capacity for learning make them valuable in a wide range of applications, including pattern recognition, forecasting, optimization, and control of complex systems. There are numerous existing methods available to effectively model complex systems. Iqbal et al. conducted an analysis of a computer virus epidemic model using fractional order differential equations^2^. Iqbal et al. also conducted an analysis on the stochastic form of the Newell–Whitehead–Segel equation^3^. Kazeem et al. utilized the exponential matrix algorithm, differential transformation algorithm, and Runge–Kutta method to simulate temperature distribution in heating tanks^4^. Liaqat et al. introduces a conformable Shehu transform decomposition method (CSTDM), a novel algorithm for solving quantum mechanical models with high accuracy and efficiency^5^. Shahzad et al. investigated fluid flow through double disks, considering various boundary conditions and incorporating the effects of microorganisms and thermal parameters^6^. Another study presents a mathematical model of non-integer order through the fractal fractional Caputo operator to analyze the development of Ebola virus infections^7^.

There are also numerous existing works where neural networks have been utilized to analyze and understand complex systems. Basma et al. introduces the numerical performances of the fractional kind of food supply (FKFS) model using fractional derivatives and stochastic scaled conjugate gradient neural networks^8^. Sabir et al. introduced a stochastic solver based on the Levenberg-Marquardt backpropagation neural networks (LMBNNs) for the nonlinear host-vector-predator model^9^. The COVID-19 spreading model is investigated using artificial neural networks with Levenberg-Marquardt backpropagation training by Umar et al^10^. Another work by Umar et al. introduces a numerical computing technique using artificial neural networks optimized with particle swarm optimization and active-set algorithms to solve the nonlinear corneal shape model^11^.

Neuroevolution is the term used to refer to the process of applying evolutionary algorithms for the optimization of neural networks^12,13^. Recent years have garnered the attention of researchers in the possibilities of integrating the search power of evolutionary computation (EC) with the learning capabilities of artificial neural networks (ANN) for a variety of applications^14^. Traditionally, Backpropagation (BP) has been the most common algorithm used to train multilayer feed-forward neural networks. As a means of reducing the network’s error, BP employs a gradient descent rule. However, BP has certain constraints. Since it excels mainly at exploiting the existing solution, it tends to converge to local optima, which might lead to subpar classification accuracy. It also has issues with both convergence speed and scalability^15^. Researchers are increasingly adopting metaheuristic algorithms with global search capabilities to build optimum weights and biases in ANNs, since these can surpass the limitations of conventional approaches. GA, a form of search heuristic, takes its cue from Darwin’s theory of evolution through natural selection, in which only the strongest and most adaptable survive to pass their genes on to future generations^16^. Er and Liu suggested a hybrid approach employing GA with BP to improve MLP Neural Network (MLPNN) parameters in 2009^17^. Singh and De developed an MLP-GA-based approach to incoming traffic in the year 2017, with the intention of detecting application layer DDoS attacks^18^. A strategy for optimizing the hyper-parameters of an MLPNN through a GA was proposed in 2018 by Itano, Sousa, and Hernandez^19^. In 2020, Ecer et al. put forward an approach to predict stock price movement direction through integrated MLP methodologies, multilayer perceptron-genetic algorithm (MLP-GA) and multilayer-particle swarm optimization (MLP-PSO)^20^. In^21–26^, training an MLP with GA and comparing its findings to those obtained via BP were carried out. Training ANN algorithms is a computationally expensive task, thus limiting its usage in processing big and complex problems. Therefore, it is desirable to implement ANNs on a parallel or distributed platform to boost performance. Calvert proposed a technique in 2005 to anticipate and evaluate the performance of distributed ANN algorithms by looking at how well they handle the relatively straightforward mathematical operations required to build the network^27^. To improve the accuracy of identifying students with learning disabilities, Wu et al. suggested parallelizing and optimizing a genetic-based ANN classifier in 2010^28^. In 2012, Casas suggested a method for parallelizing the backpropagation algorithm used to train a network that predictions the SP 500 Index. Parallelizing the backpropagation technique to operate on four processors concurrently reduced training time by 61%^29^. In 2012, Gonzalez et al. proposed a multi-step ahead TSF (Time Series Forecasting) using a fully automatic Evolutionary ANN (EANN) system using two parallel programming standards: Message Passing Interface (MPI) and Open Multi-Processing (OpenMP)^30^. In this study, a distributed processing framework for GA-evolved neural network classifier has been explored, as well as the effectiveness of this framework in big data classification problems. A comprehensive review and recent advances of EC in ANN and other machine-learning algorithms can be found in Telikani et al.^31^.

## Distributed evolutionary neural network

### Overall architecture

**Figure 1:**
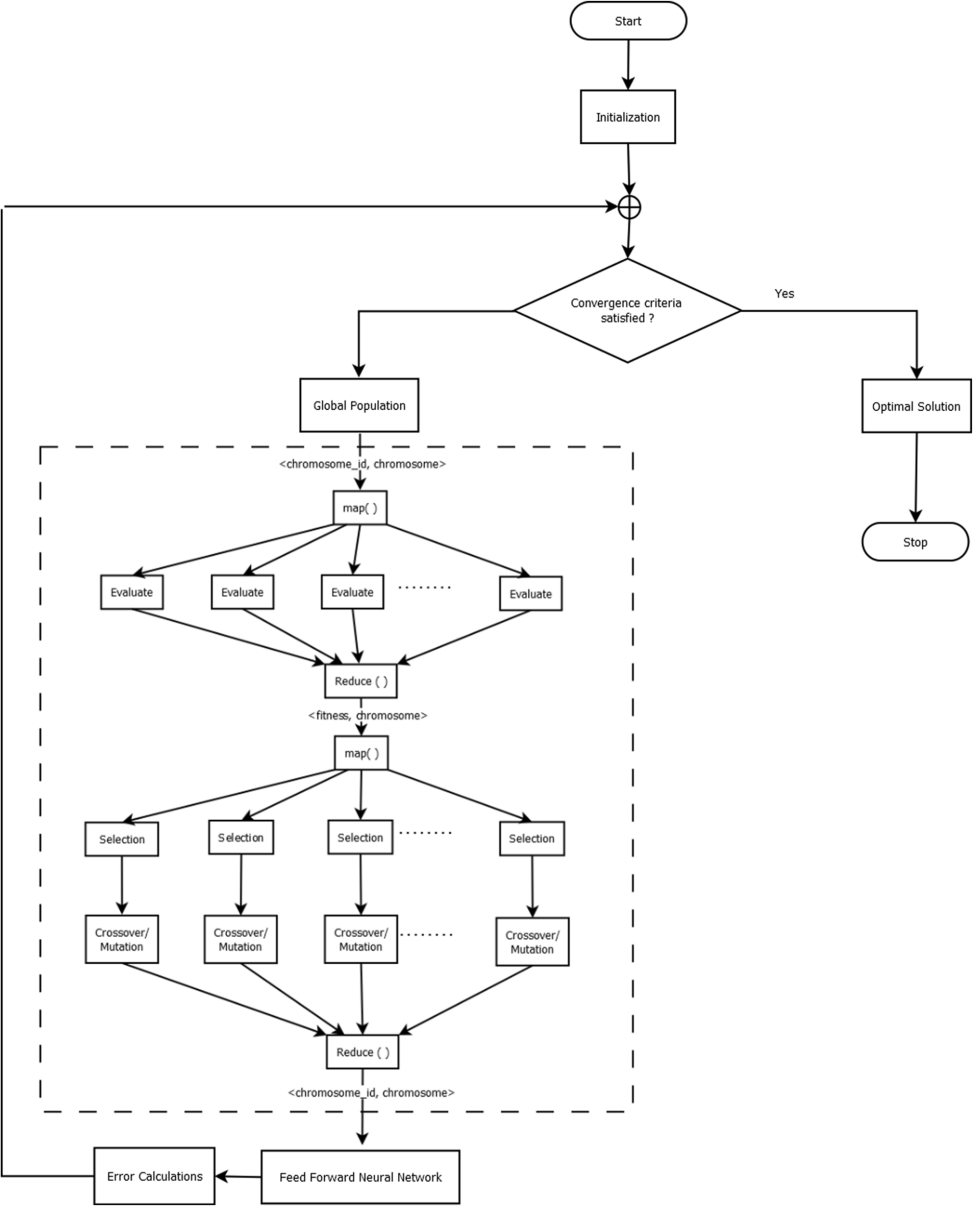
Overall architecture of proposed distributed evolutionary neural network.

This study proposes a metaheuristic method to train the neural networks (Fig. 1). GA is used to determine suboptimal values of weight coefficients and bias for the Artificial Neural Network. With an input layer, two hidden layers, and an output layer, our ANN model has a total of four layers. A distributed GA Architecture is employed to train the neural networks to improve the model’s effectiveness in handling large datasets.

### Training neural networks with GA

Weight training for an ANN using a GA entails three stages. The first step is to determine the chromosome representation of connection weights for the GA population; the second step is to evaluate the fitness of these connection weights by testing the efficacy of the weights in an ANN and computing the mean square error; and the third step is to apply the evolutionary process of GA, which includes selection, crossover, and mutation. Evolution ceases when fitness exceeds a specified value or the population converges. The Genetic Learning of ANN weights can be described using the following stages:

(i) Each chromosome in the population represents the weight matrix of the neural network. The population is initialized in such a way that each chromosome is initialized with a random set of integers as genes. The population size and size of a chromosome is initialized as hyper-parameters.
(ii) Evaluate each set of the connection weights by passing them through the feed-forward ANN model to make a prediction and compute the total mean square error between the predicted and target outputs. Total MSE (Equation (1)) is used to estimate the fitness of an individual chromosome.
  1 $$\begin{aligned} MSE = \sum _{i=1}^{n}(Y_{i}-\hat{Y_{i}}) \end{aligned}$$
(iii) In the next phase, based on their fitness values, the best chromosomes are selected as parents for reproduction in the new population. This aids the model to converge or move to optimal solutions. A selection method based on a roulette wheel is adopted^16^. The population of the current generation is represented on a roulette wheel, with each chromosome occupying a slot in proportion to its fitness.
(iv) The crossover and/or mutation operators are then applied to parent chromosomes to generate offsprings and increase genetic variability, forming the next generations. This study uses a single-point crossover, in which a random combination point is chosen for both parents’ chromosomes. The chromosomal segments after these combination locations are exchanged, producing two new offsprings. After applying crossovers, mutation is applied on randomly selected chromosomes based on the mutation rate^32^. If the stopping condition is met the fittest individual in the population is returned; otherwise, the evolution continues.

### Distributing genetic operators using map reduce

This section describes the distribution of genetic operators of GA for weight training of ANN as depicted in Fig. 1. The data is split into 80% for training and 20% for testing purposes. The proposed Apache Spark is utilized to distribute the GA. Distributed GA generates an initial population of solutions at random and distributes them as an RDD across multiple partitions. Using the parallelize method, the initialized population is parallelized into populationRDD and then divided into segments, with each segment being assigned to a separate node in the distributed environment for processing. After mapping the parallel population segments with a fitness function, each segment of the population is evaluated in parallel. The fitness of each particle in the population is calculated using mean-squared error. The fitness value of each chromosome is evaluated on different workers where the chuck of the population that has the particular chromosome resides. Following that, the driver program collects the results and performs genetic operators on them. In the context of the spark driver execution, the parallelize() method of Spark is used to convert the initial population into a populationRDD that comprises pairs of chromosome identifiers and their corresponding chromosomes, < chromosome_id, chromosome>. The training data is partitioned across the nodes using the parallelize() method. After that, a map transformation of spark is executed as map(evaluateFitness()) is applied to the populationRDD and the training data to compute the fitness score of each chromosome in the population and to turn populationRDD into fitnessValueRDD, which contains pairs of <fitness, chromosome> entries. The training data is distributed across the nodes into different partitions, and the driver program executes the evaluateFitness() function on the cluster in parallel on different worker nodes to compute the fitness values of each partition of the training data. When the evaluation phase concludes, and the collect() operation initiates the collection of these pairs to the driver. Next, the fitnessValueRDD is parallelized using the parallelize() function of Spark where it is divided into subpopulations. Then the mapPartitions(geneticEvolution()) transformation, is invoked where the geneticEvolution() function is performed in parallel on each worker node which contains different partitions of the fitnessValueRDD. In the function geneticEvolution() three genetic operators are applied, selection(), crossover() and mutation() functions and a evolutionRDD is produced in the end, which contains the evolved chromosome <chromosome_id, chromosome> pairs. In selection(), a roulette wheel selection mechanism is used to select the best individuals for reproduction in the next generation, crossover() uses the selected best individuals from selection() for reproduction and produces better offspring and mutation() is then performed on the produced offsprings so that the model converges to a solution faster. To perform crossover, all the selected chromosomes are sampled and stored into two even list RDDs, which are parallelized using the parallelize() function and key-value pairs of two random chromosomes in the two lists are formed. A map() function is used to perform single-point crossover over the key-value pairs, <chromosome, chromosome>, one by one and the crossoverRDD is produced. After the crossover is complete, the gene loci of randomly selected chromosomes are traversed using map() with the mutation factor specified as a hyperparameter, and then a negation operation is done on the gene loci to produce a new chromosome. As a result of mutation, the evolvedRDD is generated and saved in the system’s memory. Once all the genetic operators have been applied, and evolvedRDD is produced, the map(.best_chromo()) transformation is applied to convert evolvedRDD into bestChromosomeRDD, which is a <key,value> pair, where the key is the chromosome_id and value is the fitness value of the fittest chromosome in the partition. At last, the collect() method compiles the top performers from each worker to determine the optimal solution, which is then used to train the feed-forward neural network.
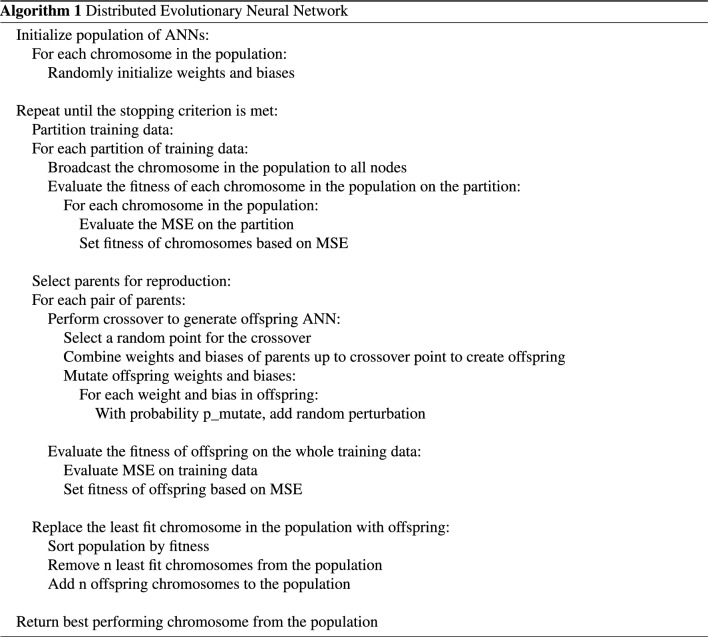


### Coordinated learning process

The GA generates numerous potential solutions to the issue at hand and then refines them over the course of several generations. Every solution contains all of the parameters that might contribute to producing better results. When applied to ANN, weights in each layer contribute to achieving high accuracy. As a result, a single solution obtained using GA will contain all of the weights used by ANN. Our ANN model consists of a total of four layers, which are comprised of an input layer, two hidden layers, and an output layer.

## Implementation and tools

The experiment was carried out on a high-performance Hadoop cluster consisting of one Name node server and two Data node servers with a total of 768GB RAM and 144 core processor. Apache Spark 3.3.0 is supported by the cluster. Apache Spark is a free and open-source distributed data processing engine that is scalable, rapid, and allows massive data processing established by UC Berkeley, which permits distributed application developers to program in Java, Python, Scala, and R. PySpark is its Python API. Not only does it provide the Python APIs needed to develop Spark applications, but it also gives access to the interactive PySpark shell for analyzing the data in a distributed environment.

## Results and discussions

### Benchmark datasets

Three datasets, HEPMASS, SUSY, AND HIGGS, are used to evaluate the performance of the proposed Distributed Evolutionary Neural Network. The datasets were obtained from the UCI Machine Learning Repository. The HEPMASS data set contains sophisticated physics experiments designed to search for exotic particles as well as a bi-classification task. The “SUSY” data discriminate between a signal process that generates super-symmetric particles and a background activity that does not correspond to the occurrence. Data referred to as “HIGGS” are samples of signals used to assess if they are consistent with the emission of Higgs Bosons. Each data set’s properties are displayed in Table 1.

**Table 1:** Details of datasets.

Dataset	Number of instances	Number of attributes	Size (GB)
SUSY^33^	5,000,000	18	2.23
HEPMASS^34^	10,500,000	28	4.82
HIGGS^35^	11,000,000	28	5.74

### Performance evaluation of the proposed model with the existing models

The performance of the model is evaluated using various performance metrics. These performance measures considered are accuracy and area under ROC. Accuracy is the degree to which the projected value closely matches the actual value. The outcome of a data point might be True Positive, TP (label and prediction are both positive), False Positive, FP (the label is negative, but the prediction is positive), True Negative, TN (label and prediction are both negative) and False Negative, FN (the label is positive but prediction is negative). The accuracy metric is described as follows:

2 $$\begin{aligned} A = \frac{(TP+TN)}{(TP+TN+FP+FN)} \end{aligned}$$

Table 2 depicts the comparison of accuracy values obtained for GA-based ANN, which works in a normal mode, and the proposed distributed evolutionary neural network.

**Table 2:** Accuracy comparison.

	SUSY	HEPMASS	HIGGS
BP-ANN	69.33	72.78	59.24
ACO-ANN	74.62	86.5	60.27
PSO-ANN	77.69	89.13	62.88
ABC-ANN	73.25	88.71	63.9
GA-ANN	76.54	90.16	64.25
DENN	78.63	90.69	67.30

Both GA-ANN and DENN give comparable results in terms of accuracy values, with DENN producing slightly better performance. It can be observed that the effect of the distributed environment does not have a negative impact on the performance of accuracy but, rather, leads to a marginal improvement in the accuracy values.

**Figure 2:**
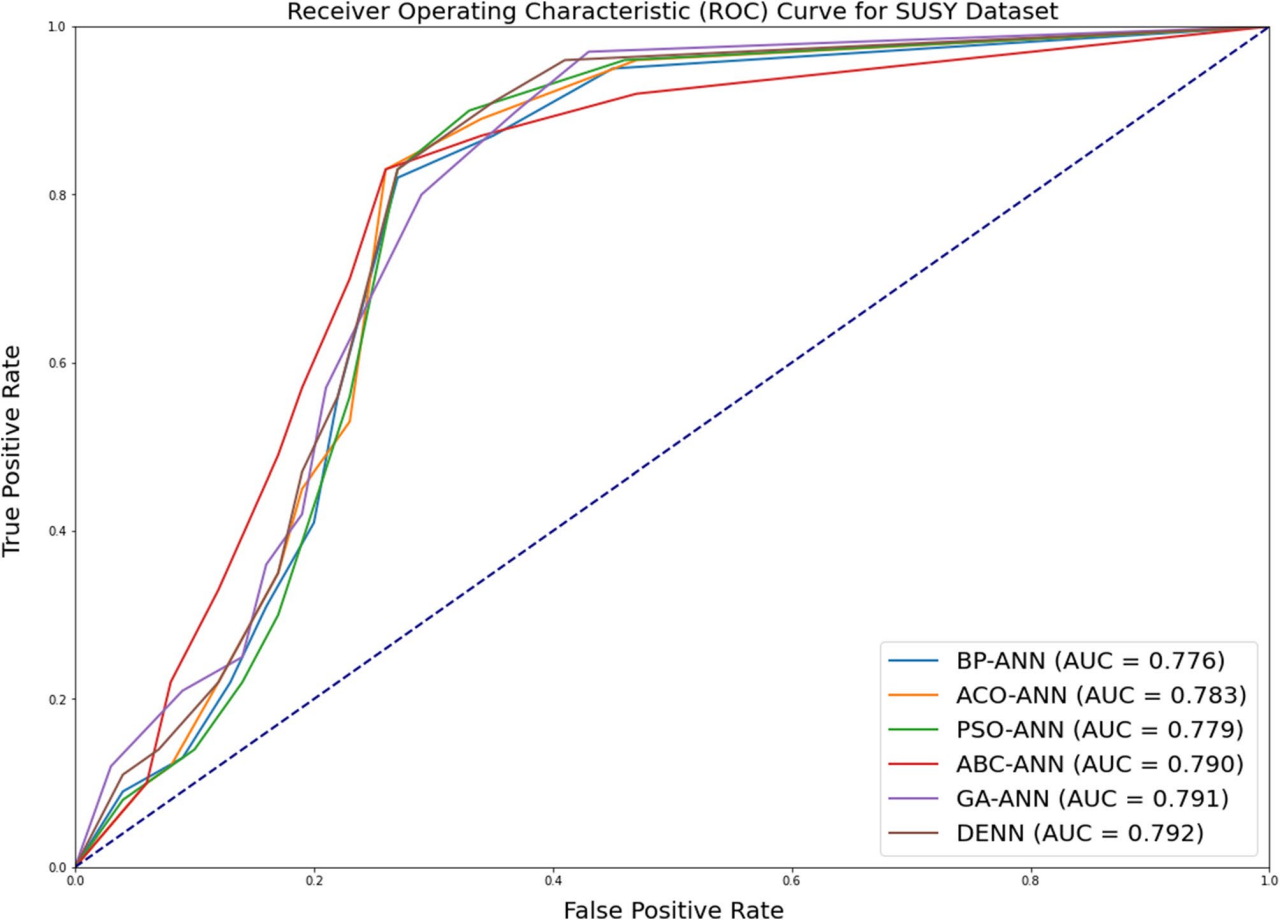
Comparison of ROC curve for SUSY dataset for all alogrithms under consideration.

**Figure 3:**
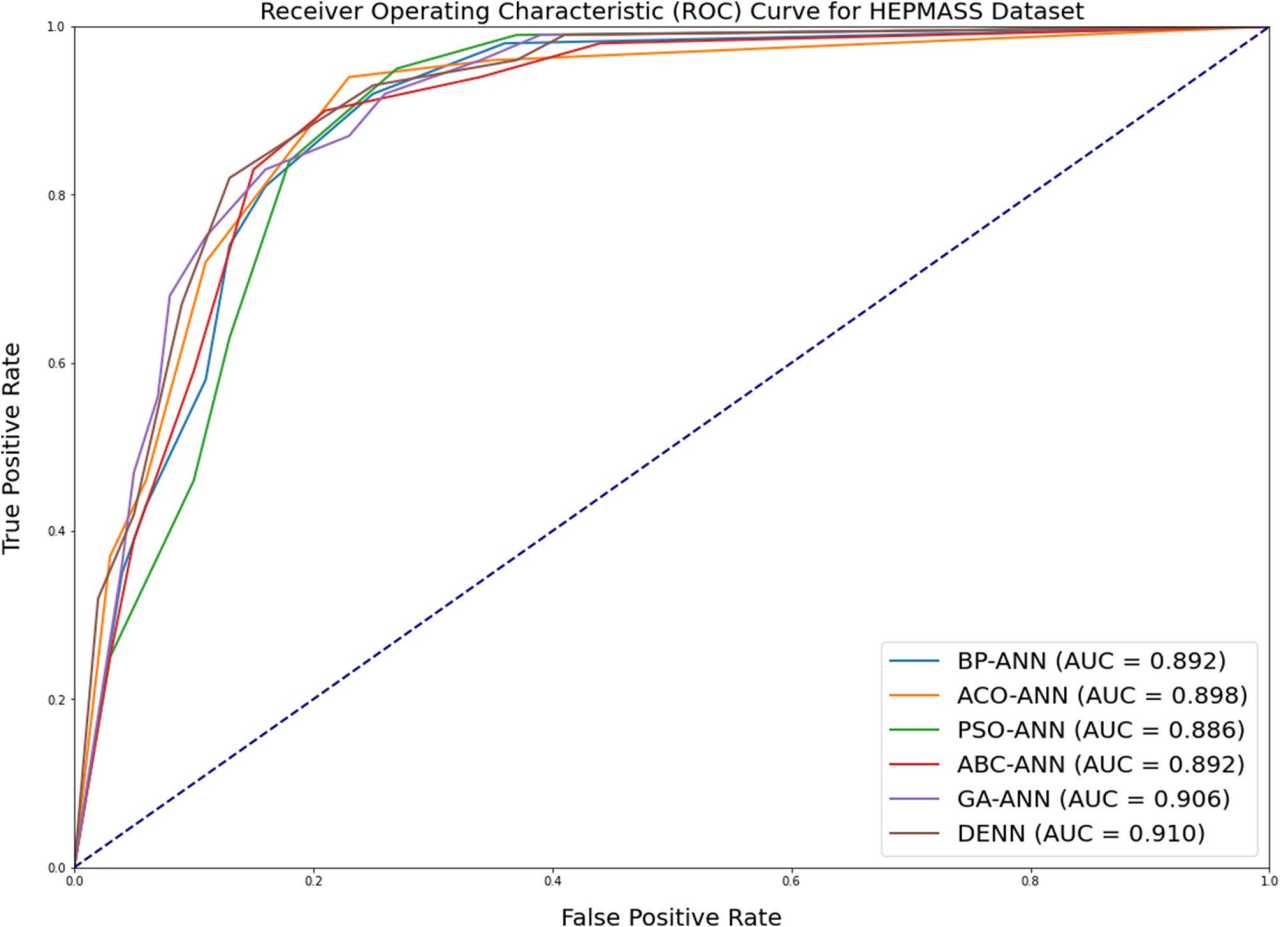
Comparison of ROC curve for HEPMASS dataset for all alogrithms under consideration.

**Figure 4:**
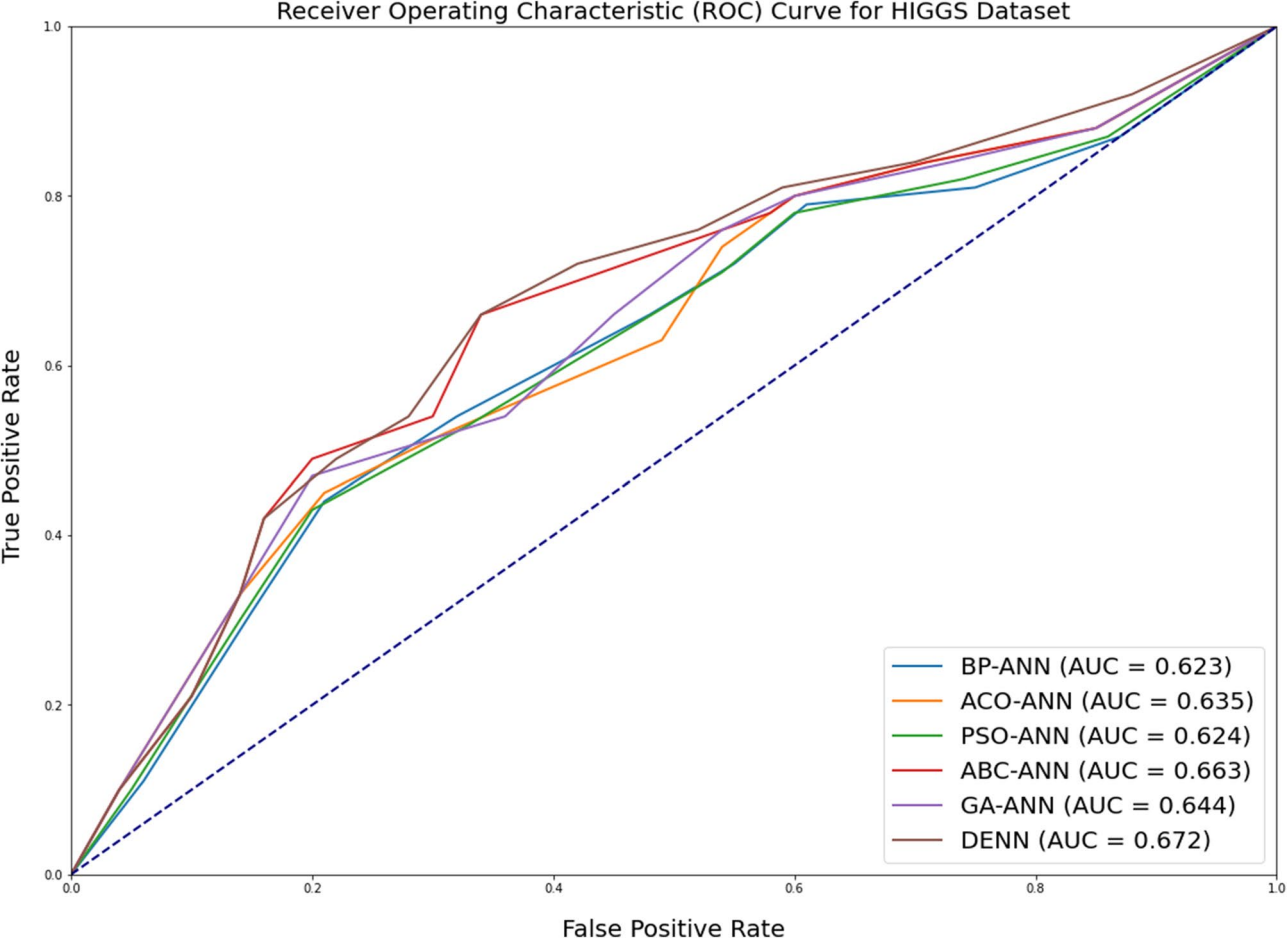
Comparison of ROC curve for HIGGS dataset for all alogrithms under consideration.

In addition to measuring accuracy, the area under ROC (Receiver Operating Characteristic) curve is assessed as well. The receiver operating characteristic (ROC) plot compares the true positive rate (TPR) to the false positive rate (FPR) at various thresholds for classifying the data. The area under the receiver operating characteristic curve (ROC) is a metric that depends on how effectively the classifier can differentiate between the two binary classes. The area’s value varies from 0 to 1. The greater the ROC area, the more accurate the prediction. Figures 2, 3 and 4 depicts the results of ROC curve and the Area under ROC curve values for all the algorithms under consideration.

As with the case with accuracy, it can be observed that there is just a marginal improvement in the case of Area under ROC Curve metric values. This depicts that the distribution does not considerably impact the accuracy or AUC measures. More Area under ROC values (usually greater than 0.65) indicates classification confidence.

### Impact on training time

Taking into consideration the execution time, it can be observed that the use of the distributed environment in DENN accelerates the learning performance of the classifier by more than 75% in all the datasets considered when compared with GA-ANN executed in normal mode (Table 3). These results were obtained when the whole of the dataset was considered for classification.

**Table 3:** Time taken comparison (in seconds).

	SUSY	HEPMASS	HIGGS
BP-ANN	23,360	42,307	43,280
ACO-ANN	21,042	40,357	39,614
PSO-ANN	18,360	41,269	33,258
ABC-ANN	16,989	38,657	36,875
GA-ANN	17,280	36,100	37,820
DENN	2960	5050	6129

A similar result also can be seen in Table 4, where distributed environment also improves the speed by around 80% for every scenario in partial training data except for when the number of rows is 10000. For a smaller number of instances, the overhead of distributing the dataset is higher; hence a significant improvement in the execution time cannot be observed.

**Table 4:** Training time comparison for partial training data (in seconds).

No. of intanses	GA-ANN	DENN
SUSY		
10,000	19.27	17.82
50,000	186.21	32.02
100,000	369.75	69.34
HEPMASS		
10,000	37.95	15.11
50,000	194.76	35.23
100,000	384.22	79.15
HIGGS		
10,000	38.41	18.24
50,000	182.05	37.33
100,000	376.52	96.53

### Speedup trend

Another metric used to evaluate the performance of the proposed model is speedup. The increase in speed of a parallel algorithm relative to its serial equivalent is known as speedup. It is an essential method for determining the efficiency of parallel processing and the impact of parallelization. Assuming the duration of the serial algorithm (single node) is Ts and the duration of the parallel algorithm (many nodes) is Tp, the speedup can be represented using the Eq. (3).

3 $$\begin{aligned} S_{p}=\frac{T_{s}}{T_{p}} \end{aligned}$$

**Figure 5:**
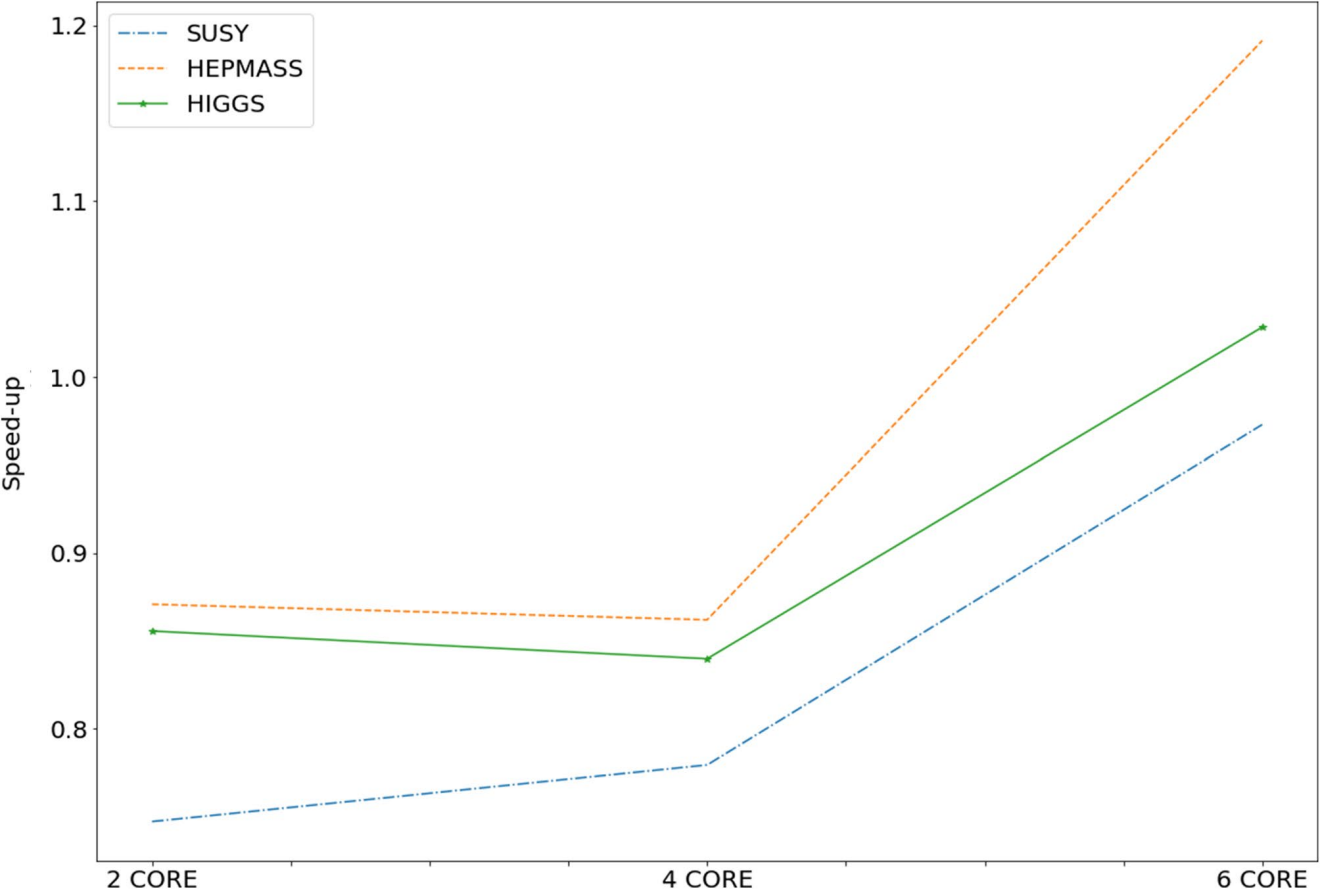
Speedup trend for three datasets considered with an increasing number of cores.

The higher the speed, the greater the parallel efficiency and performance. Figure 5 depicts the speedup trends observed for the datasets taken into consideration, SUSY, HEPMASS and HIGGS, for an increasing number of cores.

It can be observed that until four cores the speedup trend is similar for all three datasets, and after four cores HEPMASS dataset has the highest speedup factor, followed by HIGGS and then the SUSY datasets.

### Scalability analysis

Scalability is also used to evaluate the performance of the proposed model. Scalability is the ability of a system to improve performance as the number of slaves rises. When employing a parallel technique, the consumption rate of the cluster is displayed.

4 $$\begin{aligned} J=\frac{S_{p}}{p} \end{aligned}$$

Equation 4 is for scalability. Speedup is denoted by Sp, and the number of slaves by p. J is a positive integer usually less than or equal to one but as Sp could be greater than p, J can sometimes be more than one. Scalability improves as it approaches one. A parallel program’s scalability curve exhibits a diminishing trend as the number of slaves rises beyond the ideal limit. Table 5 represents the Performance analysis of the proposed model with an increasing number of cores.

**Table 5:** Performance analysis of the proposed model with an increasing number of cores.

Number of nodes	Time taken (in seconds)	Accuracy
SUSY		
2	11562.14	78.63
4	5542.22	78.62
6	2960.07	78.63
HEPMASS		
2	20726.05	90.30
4	10469.32	90.69
6	5050.12	90.69
HIGGS		
2	22100.16	67.30
4	11257.34	67.29
6	6129.4	67.30

**Figure 6:**
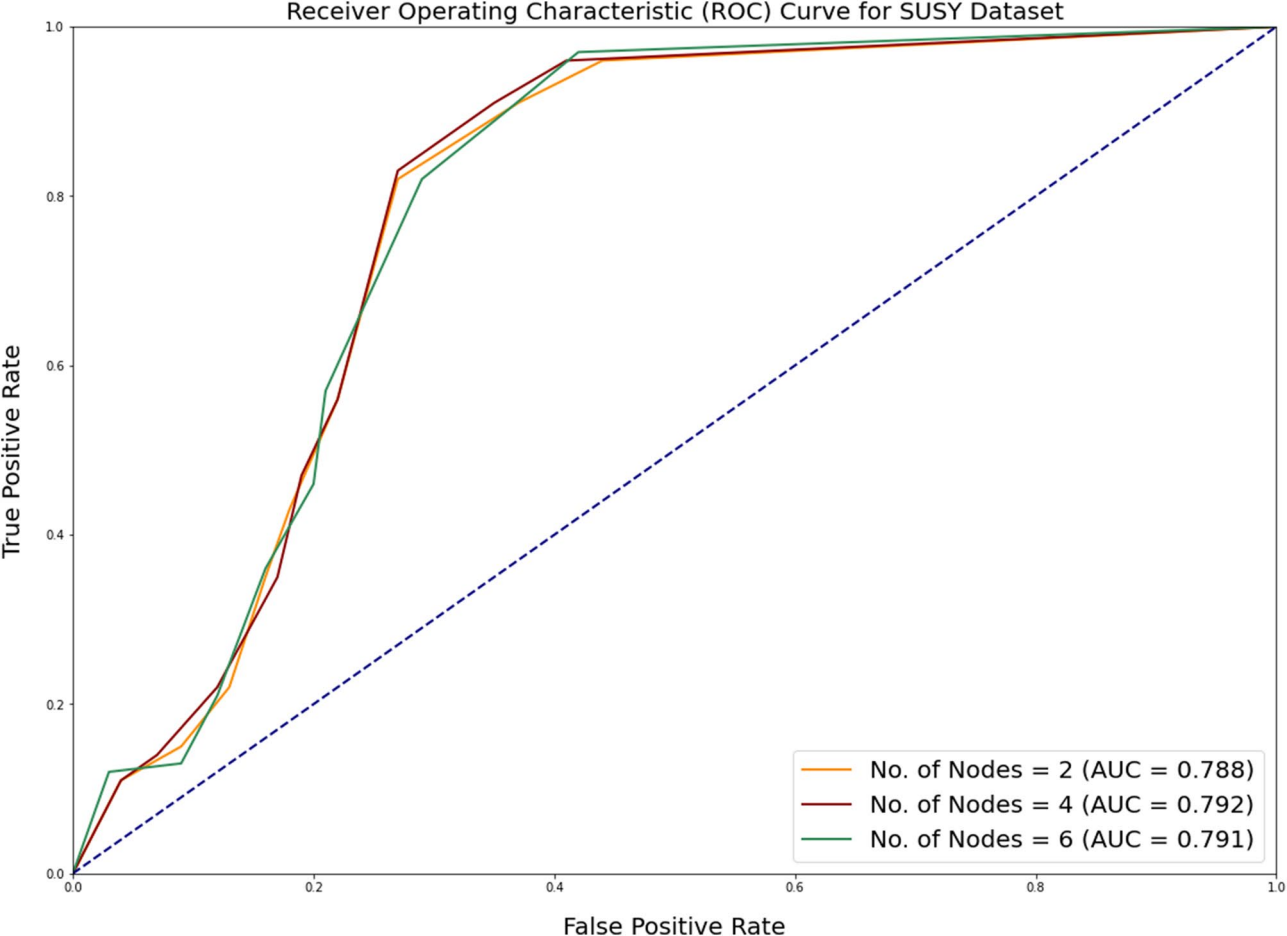
ROC curve for SUSY dataset with increasing number of cores.

**Figure 7:**
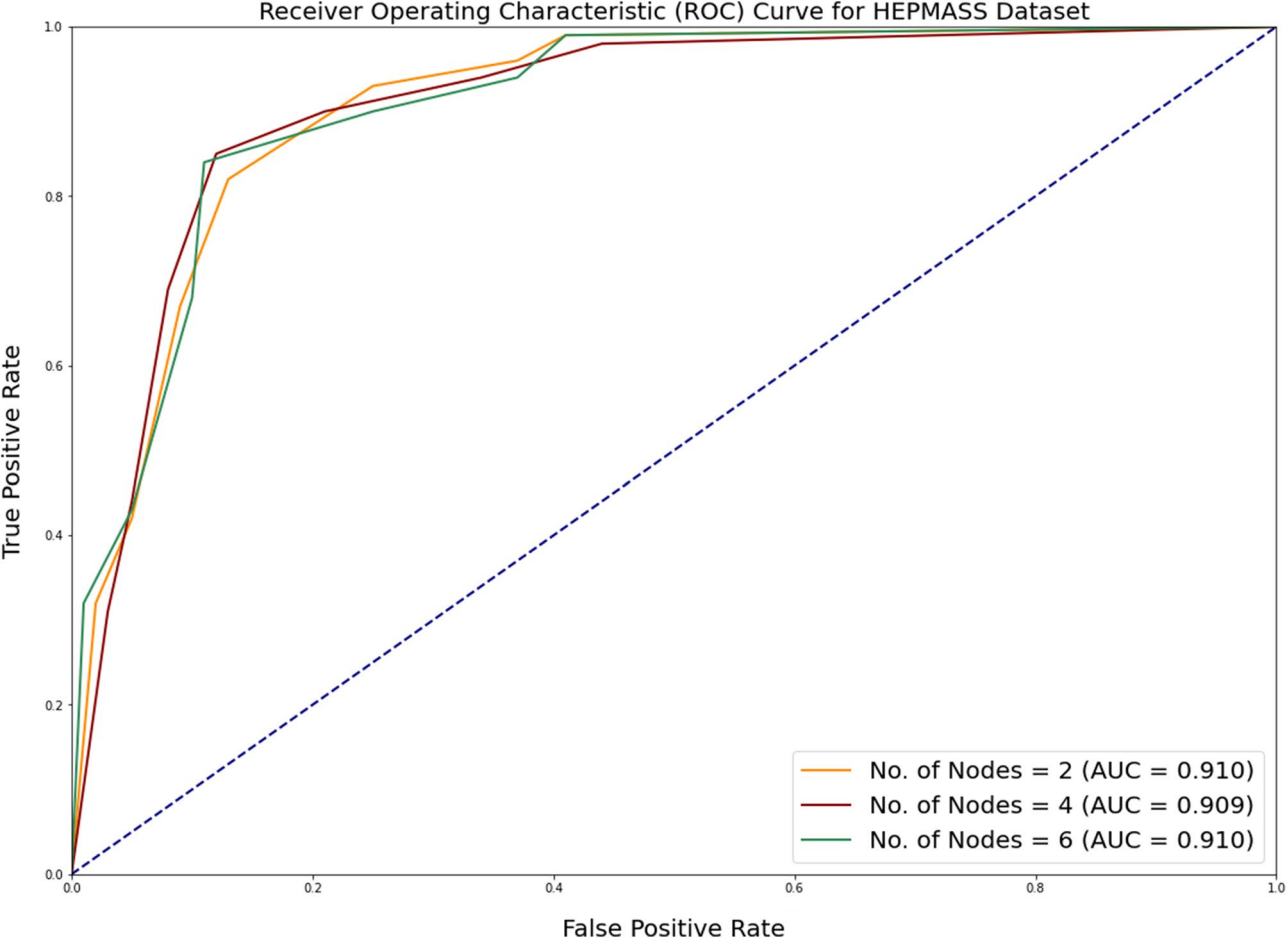
ROC curve for HEPMASS dataset with increasing number of cores.

**Figure 8:**
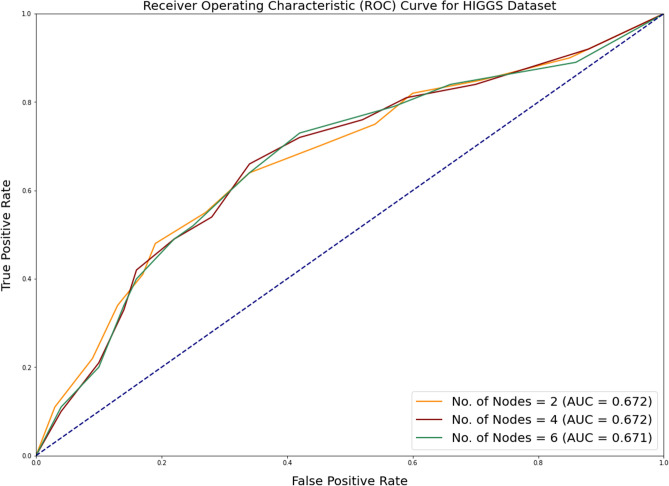
ROC curve for HIGGS dataset with increasing number of cores.

Figures 6, 7 and 8 depicts the ROC curve obtained for SUSY, HEPMASS and HIGGS dataset with respect to increasing number of cores. The ROC (Receiver Operating Characteristic) curve is an evaluation metric commonly used in classification tasks to assess the performance of a model in distinguishing between different classes. These metrics measure the model’s ability to correctly classify instances across various threshold values.

**Figure 9:**
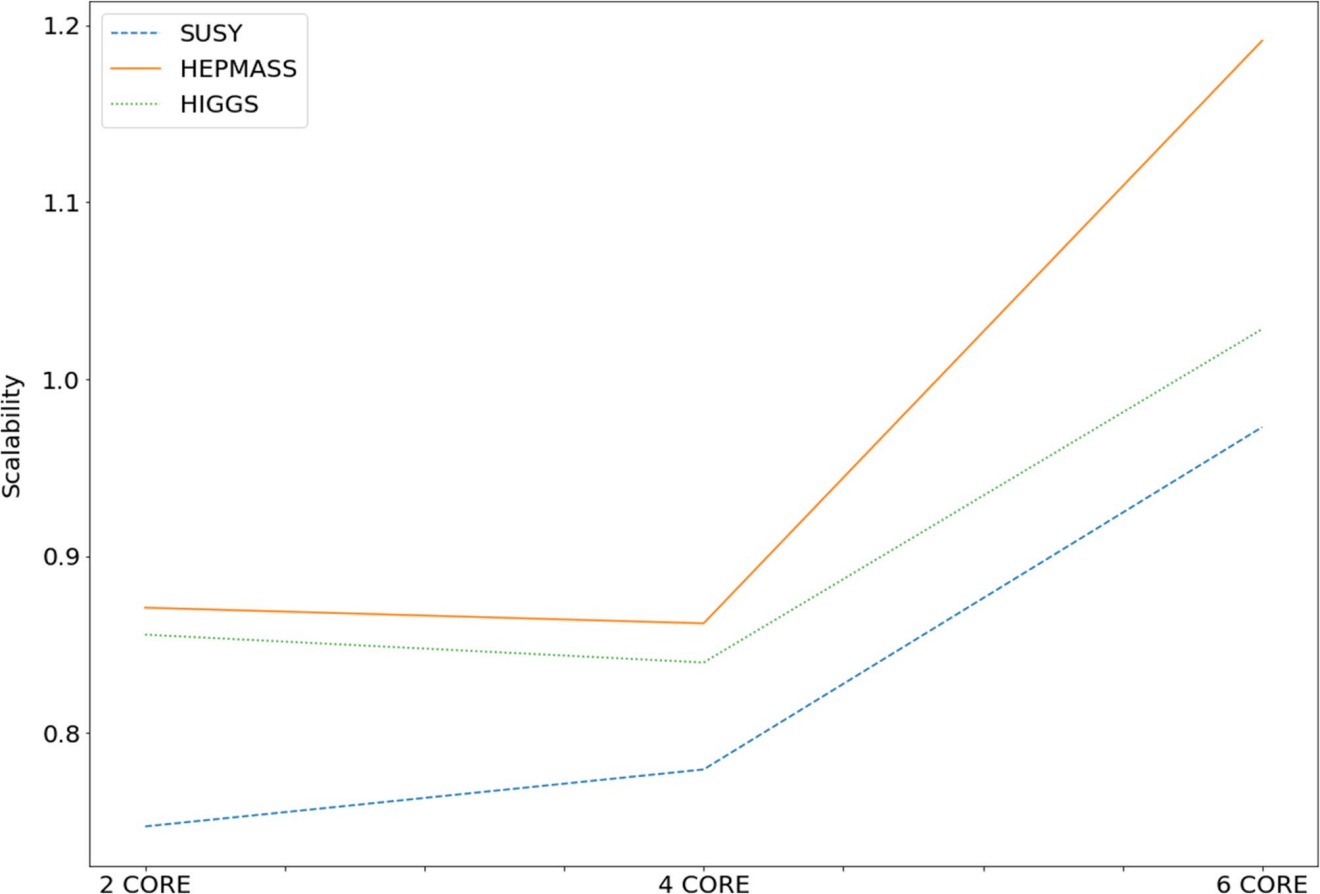
Scalability analysis of the model.

Figure 9 illustrates the scalability of our proposed model, showcasing how it improves as the number of cores increases. The graph clearly indicates that scalability continues to increase until six cores are utilized. However, beyond this point, the scalability trend begins to decline. This decline occurs when the overhead of distributing the model outweighs the benefits gained from increased efficiency in distributed processing. In essence, the graph demonstrates that there is an optimal point where further increases in the number of cores may not yield significant scalability improvements due to the associated distribution overhead.

## Conclusion

An in-depth discussion of the benefits and significant drawbacks of the ANN evolved using GA has been provided in this paper. A distributed GA model was adopted to train the neural network. Accuracy, AUC & ROC, Time Taken, Speedup and Scalability were taken as measures to evaluate the performance of the model. It is discovered that the accuracy and AUC & ROC do not considerably improve when the algorithm is executed in distributed mode but are still on par with the traditional methods. This happens due to the similarity in the implementation of the proposed method and the traditional methods. Increasing the number of nodes will affect the computation time required, but it will not change how the algorithm is performed. When the distributed GA is used, there is a prominent improvement in the execution times. The execution time improved by almost 80% in the case of most datasets. The speedup and scalability trends tend to increase as the number of cores used to distribute the model increases up until an optimum value. After that the speedup and scalability does not show considerable improvement due to the distribution overhead. The optimum number of nodes identified for the proposed model is 6 nodes. The proposed method proved the utilization of GA in a distributed paradigm significantly improved the speedup and scalability, which can also be adapted to many other learning algorithms for Bigdata.

## Data Availability

The datasets analysed during the current study are available in the UCI machine learning repository. In particular, SUSY Data Set available online at https://archive.ics.uci.edu/ml/datasets/SUSY, HEPMASS Data Set available online at http://archive.ics.uci.edu/ml/datasets/hepmass and HIGGS Data Set available online at https://archive.ics.uci.edu/ml/datasets/HIGGS.
